# Cell ontology in an age of data-driven cell classification

**DOI:** 10.1186/s12859-017-1980-6

**Published:** 2017-12-21

**Authors:** David Osumi-Sutherland

**Affiliations:** European Bioinformatics Institute (EMBL-EBI), Wellcome Trust Genome Campus, Hinxton, CB10 1SD UK

**Keywords:** Single cell, Unsupervised clustering, scRNAseq, Cell atlas, Ontology, Owl, Drosophila, Mouse, Retinal bipolar neuron, Antennal lobe projection neuron

## Abstract

**Background:**

Data-driven cell classification is becoming common and is now being implemented on a massive scale by projects such as the Human Cell Atlas. The scale of these efforts poses a challenge. How can the results be made searchable and accessible to biologists in general? How can they be related back to the rich classical knowledge of cell-types, anatomy and development? How will data from the various types of single cell analysis be made cross-searchable? Structured annotation with ontology terms provides a potential solution to these problems. In turn, there is great potential for using the outputs of data-driven cell classification to structure ontologies and integrate them with data-driven cell query systems.

**Results:**

Focusing on examples from the mouse retina and *Drosophila* olfactory system, I present worked examples illustrating how formalization of cell ontologies can enhance querying of data-driven cell-classifications and how ontologies can be extended by integrating the outputs of data-driven cell classifications.

**Conclusions:**

Annotation with ontology terms can play an important role in making data driven classifications searchable and query-able, but fulfilling this potential requires standardized formal patterns for structuring ontologies and annotations and for linking ontologies to the outputs of data-driven classification.

## Background

### Data-driven classification of cell types

Data driven classification of cell types via unsupervised or semi-supervised clustering is becoming common. Examples include classifications derived from transcriptomic profiles from single cell RNAseq [1] and seqFISH [2], from neuronal morphology [3] and neurophysiology [4]. Other methods are likely to follow with the collection of other large datasets profiling single cells including single cell metabolomic data [5] and complete connectomic profiles of cells [6, 7]. Classification from transcriptomic profiles is likely to become dominant via large scale projects including cell atlases for Humans [8] and *Drosophila* [9].

It is still an open question whether these different approaches to classification will produce multiple, orthogonal classifications, distinct from classical classifications, but early results suggest not. For example, the unsupervised classification of retinal bipolar cells using single cell RNAseq data recapitulates and further subdivides classical classifications of these cell types, rather than being consistent with a novel classification scheme [1]. Similarly, unsupervised clustering of imaged single *Drosophila* neurons using a similarity score for morphology and location (NBLAST) identifies many well-known *Drosophila* neuron types [3]. These results and others are consistent with the existence of cell types corresponding to stable states in which cells have characteristic morphology, gene expression profile, and functional characteristics etc.

### Data-driven queries for cell types

With data driven classification comes the possibility of data-driven queries for cell-types. NBLAST is already in use as a query tool allowing users to use a suitably-prepared neuron image to query for neurons with similar morphology, with results ranked, as for BLAST, using a similarity score.

BLAST-like techniques are also being developed to automatically map cell identity between single cell RNAseq experiments. For example, SCMAP [10] can map between cell clusters from two different single cell RNAseq analyses, or from clusters in one experiment to single cells in another.

Unsupervised clustering of transcriptomic profiles to predict cell-types also produces a simpler type of data that might be used for data-driven queries for cell-types: small sets of marker genes whose expression can be used to uniquely identify cell-types within the context of a clustering experiment. A clustering experiment that uses CD4 positive T-cells or retinal bipolar cells as an input may provide unique sets of markers for subtypes of these cells. Where these correspond to known markers of subtypes of CD4 positive T-cells or retinal bipolar cells they can be used directly for mapping, where not they can be used to define new cell types.

### Coping with the deluge

These new single-cell techniques hold enormous promise for providing detailed profiles of known cell types and identifying many new cell types. In combination with targeted genetic manipulation, they promise to unlock a transcriptome level view of changes in cell state and differentiation [11].

But this work faces a problem, especially when carried out on a scale as large as the Human Cell Atlas. How can the results be made searchable and accessible to biologists in general? How can they be related back to the rich classical knowledge of cell-types, anatomy and development? How will data from the various types of single cell analysis be made cross-searchable? Clearly data-driven queries for cell-type will be an important part of the solution, but to be useful to biologists, single cell data needs to be attached to human-readable labels using well-established classical nomenclature. Where new cell-types are described, we need standard ways to record the anatomical origin of the analyzed cells as well as the developmental stage and characteristics of the donor organism (age, sex, disease state *etc*).

### Classification and annotation of cell types by ontologies

We already have computer-readable representations of classical classifications of cell types in the form of cell-type and anatomy ontologies. The Cell Ontology is a (mostly) species-neutral ontology of cell types [12]. Species-specific cell-type classifications exist in in a number of single-species anatomy ontologies including ontologies of zebrafish (Zebrafish anatomy ontology [13]), *Drosophila* (Drosophila anatomy ontology [14]) and human anatomy (Foundational Model of Anatomy [15]).

Each of these ontologies provides a controlled vocabulary for referring to cell-types and a mapping to commonly-used synonyms. Each also provides a nested classification of cell-types and records their part relationships to gross anatomy. They are commonly used to annotate gene expression, phenotypes and images.

These class and part hierarchies are commonly used for grouping annotations. For example, if a gene is annotated as expressed in a retinal bipolar neuron we might use classification and part relationships in an ontology to infer that it is expressed in the retina and expressed in a (type of) neuron.

It is, of course, not always clear precisely what known cell type, if any, corresponds to a single cell whose image or transcriptome we have or corresponds to a cluster of similar cells predicted by unsupervised clustering. In this case, ontologies can be a source of more general cell classifications that may applicable (lymphocyte; cortical interneuron; epithelial cell). Along with other information, they can also be used to describe the properties of unidentified cells. For example, virtual Fly Brain records the location of the various parts of unidentified neurons depicted in single cell images on the site, as well as the transgenes they express.

Specifying context in this way can be very useful to working with the outputs of unsupervised clustering of trancriptomic data – by providing a way to specify a context within which sets of marker genes defined by this analysis can be used to uniquely identify cell-types.

Conversely, the knowledge recorded in ontologies (part relationships, developmental stage, records of function) and in annotations may also be useful in homing in on candidate mappings for unmapped single cells. For example, the Drosophila anatomy ontology has been used to record the expression of transgenes in specific neuron types in the *Drosophila* brain and to record which brain regions these neuron-types overlap. Both these types of information are recorded for individual neurons.

In as far as these ontologies accurately record nomenclature, classification and part relationships to anatomy they are ideally suited to provide a mechanism for annotation of single-cell experiments. But cell ontologies will only be able to play this role if they are sufficiently accurate, flexible and scalable enough to keep up with the flood of new data.

### Making cell ontologies scalable and query-able with design patterns

Scalability and accuracy and query-ability of ontologies depends on formalization. All except the human-specific Foundational Model of Anatomy (FMA) are expressed in Web Ontology Language 2 (OWL2). OWL2 is a description logic that allows the expression of assertions about classes (the class of all neurons) and individuals (the individual neuron depicted in Fig. 3b) using quantified logic [16]. For example, we might assert that all retinal bipolar neurons are synapsed by a photoreceptor cell, or that any neuron that secretes glutamate as part of synaptic transmission is a type of glutamatergic neuron. These types of assertions are used to automatically OWL classes in a large and increasing number of ontologies (e.g. [12, 14, 17] In some resources, such as Virtual Fly Brain (VFB), they are used to classify individuals and to drive query systems [14, 18–21].

Multiple axes of classification are required for cell ontologies to be useful to biologists: A single neuron may be classified by structure (pseudo-bipolar), electrophysiology (spiking), neurotransmitter (glutamatergic), sensory modality (secondary olfactory neuron), location(s) within the brain (antennal lobe projection neuron, mushroom body extrinsic neuron), etc. But manually maintaining these multiple axes of classification simply doesn’t scale: adding new terms requires (human) editors to know all of the appropriate classifications to add and how to rearrange existing classifications to fit the new term. It also requires them to understand the intent behind existing manually asserted classifications, which is typically partially documented at best. To cope with this, many ontologies have gradually moved over to using something approximating ‘Rector’ normalization [22]: minimizing the use of asserted classification in favor of automatically inferred classification driven by OWL equivalence axioms specifying necessary and sufficient conditions for class membership. Consistency is maintained by the use of standard design patterns for representing class properties. The same design patterns can be used to annotate individuals allowing cross-querying of the ontology and individuals and auto-classification of individuals.

This approach has been used for a wide range of ontologies including the Gene Ontology [17], the Drosophila Anatomy Ontology [14] and the Cell Ontology [12]. In the Drosophila anatomy ontology, which includes 4767 cell classes, 48% of classifications (5893/12233) are automated via 2807 equivalent class axioms. In the Cell Ontology 59% of classifications (1910/3253) are inferred based on 2907 equivalent class axioms.

The strength of this approach is that it can be used to integrate diverse types of knowledge and data into a single query-able classification. An ontology might record information about the structure, function, lineage, location, connectivity and gene expression of some class of neuron or of an individual neuron and use one or more of these properties to classify it. A potential weakness is the mismatch between quantified logic, which records assertions about *all* members of a class, and the messy, noisy reality of biology and the data we collect about it. For example, when single cell transcriptomics and unsupervised clustering are used to find and predict cell types, the same experiments identify markers that can be used to distinguish them from other cell-types identified in the same experiment. These markers could be used to formally define cell-types. But, either through natural variation, or noisy data, these markers are not perfect – all have some level of false positive and false negatives when judged against clusters mapped to cell types [1, 23].

Here I present two case studies of how formalizing cell ontologies and using them to annotate the results of single cells analysis can improve the searchability and query-ability of the single cell data. In both cases I explore how we might use the outputs of single-cell analysis to extend cell ontologies and link them to data that can be used for data-driven queries for cell types.

## Results

### Case study: Mouse retinal bipolar neurons

#### Background

Retinal bipolar cells (RBCs) are a well characterized class of neurons of that transduce and process signals from the rod and cone photoreceptor cells of the vertebrate retina. RBCs are classically divided into classes based on whether they are synapsed by rod or cone cells (and if so by which types of cone cell), which laminas of the inner plexiform layer of the retina their axons arborize in and on the morphology of their axonal arbor [24]. Mammalian RBCs can also be divided into functional groups depending on whether they depolarize in response to a light stimulus (ON) or to the removal of a light stimulus (OFF) and whether they carry chromatic or achromatic information. A complete connectome for a single region of the mouse retina provides connectomic profiles and circuit context for over 400 RBCs [7]. A classification derived from unsupervised clustering of 25,000 single mouse RPC transcriptomes by Shekhar and colleagues [1] found 15 cell types distinguishable by transcriptome. This study also identified marker genes for each cell type which they then used in microscopy studies to determine morphologies of cells corresponding to each type. This, along with mapping of previously known marker genes to transcriptomes, showed that the transcriptomic derived types recapitulated and further subdivided classical classifications.

#### Formalizing the representation of retinal bipolar neurons to enhance querying and grouping of transcriptomic data

**Fig. 1:**
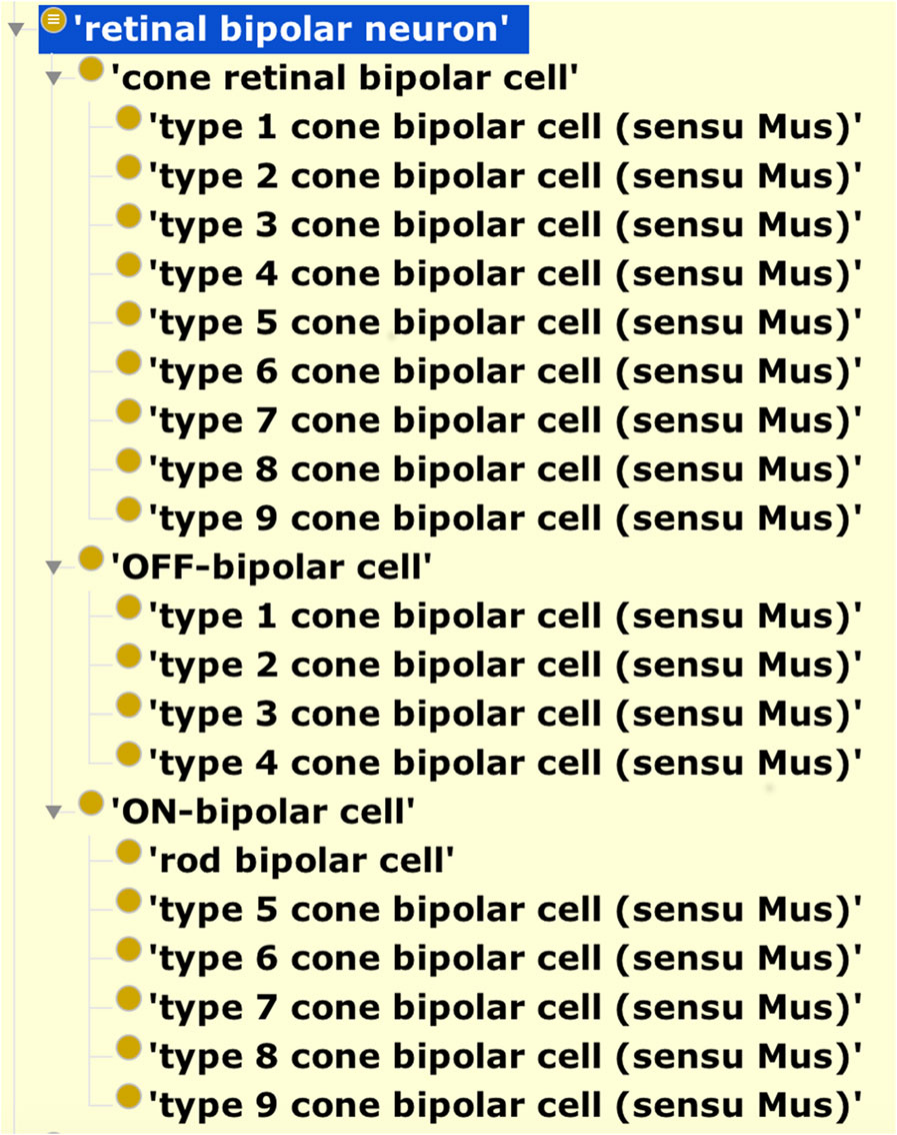
Classification of retinal bipolar cells in the cell ontology. Note that general types (rod, cone, ON, OFF) are non-species specific, whereas specific types are specified for mouse. This is necessary because morphologically defined classes vary between species

**Fig. 2:**
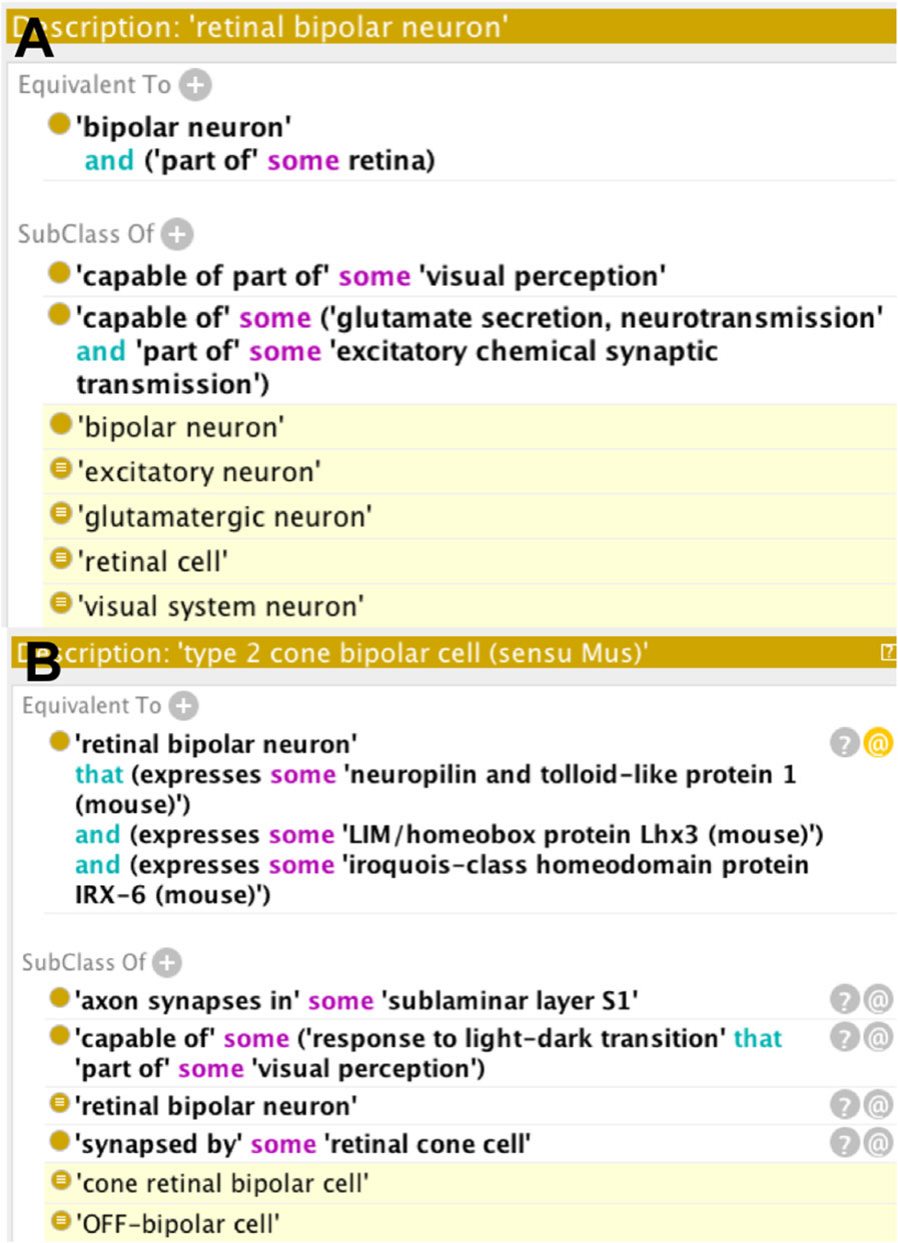
Automated classification of retinal bipolar neurons in the cell ontology. Panel **a**: Axioms linking ‘retinal bipolar neuron to GO terms (‘visual perception’, ‘glutamate secretion, neurotransmission’, ‘excitatory chemical synaptic transmission’) along with axiomatization elsewhere in CL (not shown) is sufficient for inferred classification (in yellow) as a glutamatergic, excitatory, visual system neuron. Panel **b**: Formal Definition of type 2 cone bipolar cell using marker genes. Subclassof axioms are sufficient for inferred classification of this cell type as a cone retinal bipolar cell and an OFF bipolar neuron

The cell ontology already contains terms for the major subclasses of RBCs in the mouse (see Fig. 1) along with manual classification by photoreceptor cell input (rod vs cone) and by function (ON vs OFF). However, prior to this work, these terms lacked formal definitions useful for automated classification and querying. Figure 2 shows extensions to the cell ontology which formalize classification the general RBC class (retinal bipolar neuron) and its major subclasses.

RBCs are known to be to be glutamatergic and to form excitatory synapses to their target cells. Fig. 2 shows axiomatization of the general RBC class (retinal bipolar neuron) leading to classification under glutamatergic and excitatory. The former classification is likely to correlate with the expression of genes involved in glutamate synthesis transport and secretion and so is a potentially a useful classification for cross-querying transcriptomic data. The new axiomatization also deploys standard patterns for recording sensory modality [20] to classify RBCs as visual system neurons.

To formalize classification of OFF vs ON responsive RBCs, I added new terms on the response branch of the Gene Ontology covering response to light-dark transition and response to dark-light transition. I then used these to compose formal axioms referring to the response to these transitions as part of visual perception, using these axioms to automate classification. This major functional subdivision of RBCs is likely to be reflected in transcriptomic differences and so is a potentially a useful classification for cross-querying transcriptomic data. I also used standard relationships for modelling neuroanatomy [18] to record which laminas of the plexiform layer each RBC innervates, making this information queryable.

#### Using the outputs of data driven classification to structure an ontology of retinal bipolar neurons

What outputs of transcriptomic, data driven classification might we usefully incorporate into ontologies? Assertions about marker expression are an obvious candidate. These are potentially very valuable to biologists seeking reliable markers for identifying specific neuronal classes in their experiments. If used to construct equivalent class expressions, they are also potentially useful for providing formal definitions for classes newly identified by transcriptomic analysis. They can also be useful for automated classification of cell-types from minimal data. For example, Shekhar and colleagues identify Igfn1 as a marker that distinguishes type 7 RBCs from other RBC types. On this basis, we could add an equivalent class axiom recording that any RBC that expresses Igfn1 is a type 7 RBC. Where multiple markers of a cell types are identified multiple equivalence-axioms could be added. This process of generating equivalence axioms could potentially be automated using mappings of cell ontology terms to data-derived clusters.

A closer look at the data reveals a potential problem: within clustered transcriptomes there are small numbers of cells that fail to express an identified maker, or express a marker diagnostic of another type. In the case of Igfn1 and type 7 RBCs the percentage of false positives appears very low, and may be acceptable. In other cases (Nnat in type 3B RBCs) the potential level of false positives is very high.

There are a number of possible strategies for dealing with this. Mappings could be limited to cases where the expected false positive rate is below some cut-off. Axioms could be annotated to include a record of the expected false positive rate. A more conservative approach would be, wherever possible, to generate equivalence axioms combining multiple gene expression assertions per cell type. I have taken this approach in extending the cell ontology (Fig. 2). However, with this pattern, automated classification from data will only be possible for experiments where expression of all marker genes is assayed.

### Case study: *Drosophila* antennal lobe projection neurons

#### Background

The antennal lobe of *Drosophila* is made up of 50 glomeruli, each of which receives input from a single type of olfactory receptor neuron. Each glomerulus is also innervated by uniglomerular projection neurons that carry olfactory information to higher brain centers [25].

The NBLAST algorithm [3] measures how similar two neurons are with respect to their morphology and location. Using co-registered single-cell image data for over 16,000 individual neurons, Costa and colleagues generated a matrix of pairwise NBLAST similarity scores for all neurons and then used unsupervised clustering to find potential cell types. Many of these clusters correspond to classically defined neuron types in the *Drosophila* brain, including many types of antennal lobe projection neurons [3].

In an independent study, Li and colleagues generated a classification for antennal lobe projection neurons using unbiased clustering based on transcriptome profiles from several thousand projection neurons at various stages of their development [23]. Cells for this study were isolated based on expression of a transgenic marker expression (GH146). VFB and FlyBase have extensive annotation of expression of this marker to cell types, providing one possible route to candidate terms for mapping transcriptomic clusters. This study didn’t identify single marker genes that could uniquely distinguish clusters, but rather identified broader markers.

The question of which data-type provides the most detailed classification is likely to vary with cell type. For example, automated classification from single-cell RNAseq profiling of *Drosophila* olfactory projection neurons shows that some neurons are indistinguishable at the transcriptomic level belong to different classes defined by location, morphology, lineage and odor response [23]. Their distinct odor response functions are likely to be conferred by their connectivity.

#### Formalizing the representation of drosophila antennal lobe projection neurons

The Drosophila anatomy ontology already includes richly axiomatised classes for all 50 known uniglomerular projection neurons defined by a combination of lineage and glomerulus innervated. It also includes classification of these neurons by sensory modality and neurotransmitter released. It captures the tract through which each projection neuron type projects and the higher brain regions that they innervate.

Annotation of clusters of single neuron images with the ontology terms enriches the annotated image data by linking it to formal, query-able descriptions of its relationships to gross anatomy (innervation, fasciculation). This allows, for example, queries for images of neurons in a specified tract, or that innervate one or more specified brain regions.

Li and colleagues find similarity in gene expression profiles between cells sharing the same lineage. The query-able lineage information encoded in the ontology will make it easy to explore this further. The Drosophila anatomy ontology also encodes and growing set of query-able formal assertions of neurotransmitter for each class of neuron and direct records of known synaptic connections between neuron types. With this information, it is possible to group transcriptomes of neurons by neurotransmitter to look for patterns of gene expression which correlate this, and to group transcriptomes of cells synapsed to these neurons to search for expression of relevant neurotransmitter receptors and associated proteins.

#### Using the outputs of data driven classification to structure the ontological representation of antennal lobe projection neurons

What outputs of NBLAST based clustering might we usefully incorporate into ontologies? It would be useful to provide a link to data that could be used for subsequent queries. The clustering algorithm used in this study identified an exemplar (most typical) neuron for each cluster.

Where clusters are mapped to ontology classes, the image of a cluster exemplar can serve as an exemplar for the class – serving a role similar to that of a type specimen in taxonomy. This can be used both as a visual reference for the morphology and location of the neuron type, and as a substrate for future queries with NBLAST or any other search tool that can use image data. The exemplar approach has already been used by VFB to define the boundaries of brain regions via links to image data. It may also prove useful for the outputs of other clustering methods, for example, a link from a cell-type classes to an exemplar transcriptomic profile might provide a substrate for SCMAP queries to identify clusters corresponding to the same or similar neuron types in other clustering experiments.

**Fig. 3:**
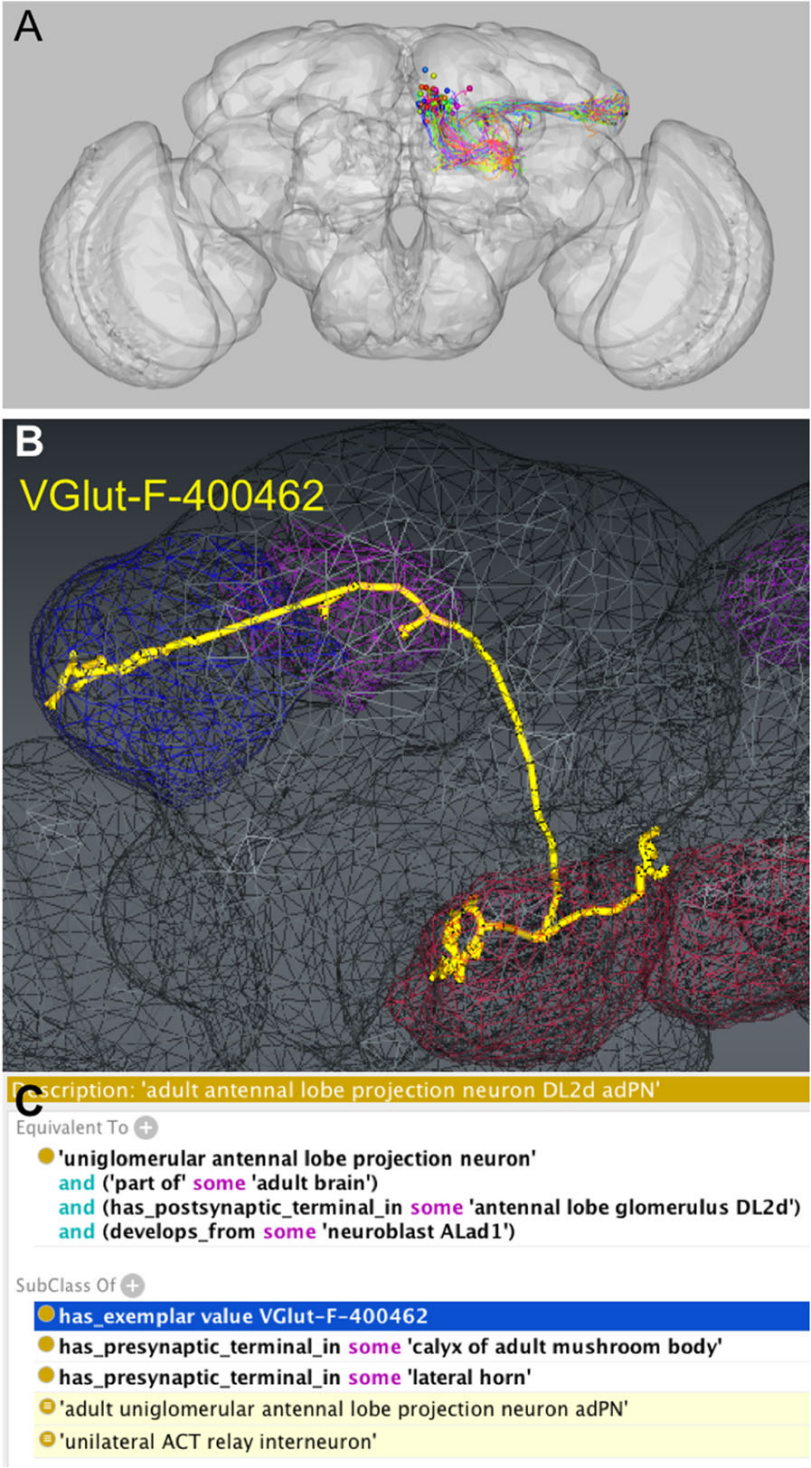
Linking projection neurons to exemplars derived from clustering. Panel **a**: Cluster of neurons with similar morphology from unsupervised clustering of >16,000 co-registered single neuron images (Costa et al. [3]) Panel **b**: VGlut-F-400462 (Chiang et al., [29]) is the exemplar (most typical neuron) from the cluster in panel A is shown in yellow. It has arborizes in the antennal lobe (AL; red), calyx of adult mushroom body (MB calyx; purple), lateral horn in (LH; blue). Image generated in VFB 2.0 alpha (unpublished). Panel **c**: OWL Axiomatization defining ‘adult antennal lobe projection neuron DL2 adPN’, which the cluster in panel **a** was manually mapped to. A minimal-commitment equivalent class axiom defines the class my lineage and innervated glomerulus. Innervation of the MB calyx and LH are recorded in subclass axioms. The axiom in blue links this class to the exemplar of the cluster, providing a standard reference for morphology and a substrate for future NBLAST queries of co-registered neurons

Figure 3, panel a shows the axiomatization of a uniglomerular projection neuron class (DL2d adPN) along with a formal link to an exemplar neuron (VGlut-F-400462) illustrated in panel b.

## Discussion

### Future challenges

The examples given here are well axiomatised, but the degree of effort put in to axiomatising will, of course, depend on use cases and resources in individual projects. Much annotation of classifications from unsupervised clusterings are likely to be simpler and more general – particularly when less well studied tissues are being characterized.

Given the huge scale of major efforts to automatically characterize and classify cell types, annotation efforts will need to be efficient and flexible. The same will apply to efforts to make use of the outputs of unsupervised clusterings to extend and refine ontology terms. For example, efficient mapping of markers to cell types would require semi-automated pipelines that can run as soon as mappings are generated. It should be possible to use machine learning methods to determine the most informative set of markers to use in classification of each cluster in the context of a single clustering analysis.

### Patterns of axiomatization

Equivalence axioms are now widely used within biomedical ontologies as a means of automating classification both within ontologies and of individuals. The success of this effort depends on devising equivalent class axioms with the minimal commitment necessary for correct classification and using standard design patterns. With this approach, it is possible for ontology editors to keep track of the basic properties and patterns needed to drive classification.

The rise of complete profiles of cell types poses some dilemmas for this approach. If, as seems likely, there are multiple sets of criteria that can be used to distinguish cell types, should this be reflected in the use of multiple equivalence axioms? To what extent should we record additional properties of classes as simple subclassing axioms? The combination of equivalence and subclassing (restriction) axioms generates hidden General Class Inclusion axioms – logically associating sets of properties with each other in a way that can be hard to keep track of.

Thomas Gruber’s ‘principle of minimal commitment’ [26] is particularly relevant to this discussion. This principle suggests that:

*“An ontology should require the minimal ontological commitment sufficient to support the intended knowledge sharing activities. A shared ontology need only describe a vocabulary for talking about a domain whereas a knowledge base may include the knowledge needed to solve a problem or answer arbitrary queries about a domain.”*

The examples in this paper illustrate how knowledge embedded in ontologies can enrich querying of datasets that provide ‘omics profiles of cell types. But we need to avoid bloating ontologies with information that allows ‘arbitrary queries about a domain’, especially where such queries could better be served via queries of annotated data. For example, while it may be useful to include qualitative assertions about marker gene expression in ontologies, arbitrary queries for cell types by gene expression should involve direct queries of transcriptomic data. Devising strategies to keep this balance sustainable will be one of the major challenges for the future development of cell ontologies.

### Linking ontologies to data-driven queries

Where ontology annotation provides broad contextual information about an individual cell-type identified by unsupervised clustering, it serves to narrow down the input data to a data-driven query for similar cell types. This is important because data-driven querying can be very compute-intensive [3, 10] making scaling across a growing dataset potentially limiting. Where more precise annotation of cell-type is possible, linking cell-types to data that can be used in data-driven queries can help users find potential matches and is potentially a source of automated annotation.

## Conclusions

Annotation with ontology terms can play an important role in making data driven classifications searchable and query-able. This role requires attention to both the lexical and formal aspects of ontology development. Extensive synonym collection is necessary to maximize findability. Formalization is needed to support multiple inheritance classification querying and automated classification of individuals from annotation. Successful formalization requires the development of clear, well documented design patterns in which equivalent class axioms are kept minimal – with clear aims in mind for use.

By supporting general assertions about cell-types and their properties, ontologies and the application of standard design patterns to annotation can support the description of single cell data at multiple levels of precision, depending on available data. This can be used to specify the context in which marker genes uniquely identify a cell type, or to provide lists of candidate cell-types for mapping to a single cell or predicted cell type from data-driven classification.

The relevance and usefulness of annotation with ontologies can be increased by suitable strategies for linking ontology term to data useful for data-driven queries for cell type.

## Methods

Ontology editing was carried out using Protégé 5.2 [27]. Ontology reasoning used the ELK OWL reasoner [28].

## Abbreviations

FACS: Fluorescence-activated cell-sorting RNAseq RNA sequencing
RBC: Retinal bipolar cell
SCMAP: Single cell map
SeqFISH: Sequential fluorescent in situ hybridization

## References

[1] Shekhar K, Lapan SW, Whitney IE, Tran NM, Macosko EZ, Kowalczyk M (2016). Comprehensive classification of retinal bipolar neurons by single-cell Transcriptomics. Cell.

[2] Shah S, Lubeck E, Zhou W, Cai L (2016). In situ transcription profiling of single cells reveals spatial Organization of Cells in the mouse hippocampus. Neuron..

[3] Costa M, Manton JD, Ostrovsky AD, Prohaska S, Jefferis GSXE (2016). NBLAST: rapid, sensitive comparison of neuronal structure and construction of neuron family databases. Neuron..

[4] Baden T, Berens P, Franke K, Román Rosón M, Bethge M, Euler T (2016). The functional diversity of retinal ganglion cells in the mouse. Nature.

[5] Zenobi R (2013). Single-cell metabolomics: analytical and biological perspectives. Science.

[6] Ohyama T, Schneider-Mizell CM, Fetter RD, Aleman JV, Franconville R, Rivera-Alba M (2015). A multilevel multimodal circuit enhances action selection in drosophila. Nature.

[7] Helmstaedter M, Briggman KL, Turaga SC, Jain V, Seung HS, Denk W (2013). Connectomic reconstruction of the inner plexiform layer in the mouse retina. Nature.

[8] Human Cell Atlas [Internet]. [cited 28 Jun 2017]. Available: https://www.humancellatlas.org/

[9] Fly Cell Atlas. In: FLY CELL ATLAS [Internet]. [cited 28 Jun 2017]. Available: http://flycellatlas.org

[10] Kiselev VY, Hemberg M. Scmap - a tool for unsupervised projection of single cell RNA-seq data. bioRxiv. 2017:150292. 10.1101/150292.

[11] Adamson B, Norman TM, Jost M, Cho MY, Nuñez JK, Chen Y (2016). A multiplexed single-cell CRISPR screening platform enables systematic dissection of the unfolded protein response. Cell.

[12] Diehl AD, Meehan TF, Bradford YM, Brush MH, Dahdul WM, Dougall DS (2016). The cell ontology 2016: enhanced content, modularization, and ontology interoperability. J Biomed Semantics..

[13] Van Slyke CE, Bradford YM, Westerfield M, Haendel MA (2014). The zebrafish anatomy and stage ontologies: representing the anatomy and development of Danio Rerio. J Biomed Semantics..

[14] Costa M, Reeve S, Grumbling G, Osumi-Sutherland D (2013). The drosophila anatomy ontology. J Biomed Semantics.

[15] Rosse C, Mejino JLV. The Foundational Model of Anatomy Ontology. In: Burger A, Davidson D, Baldock R. (eds) Anatomy Ontologies for Bioinformatics. Computational Biology. London: Springer. 2008;6. doi:10.1007/978-1-84628-885-2_4.

[16] Hitzler P, Krötzsch M, Parsia B, Patel-Schneider - W3C … PF, 2009. OWL 2 web ontology language primer. w3.org. 2009; Available: https://www.w3.org/TR/2009/PR-owl2-primer-20090922/all.pdf

[17] Mungall CJ, Dietze H, Osumi-Sutherland D. Use of OWL within the gene ontology. In: Maria Keet C, editor. Proceedings of OWLED 2014, vol. 2014. p. 25–36.

[18] Osumi-Sutherland D, Reeve S, Mungall CJ, Neuhaus F, Ruttenberg A, Jefferis GSXE (2012). A strategy for building neuroanatomy ontologies. Bioinformatics.

[19] Milyaev N, Osumi-Sutherland D, Reeve S, Burton N, Baldock RA, Armstrong JD (2012). The virtual fly brain browser and query interface. Bioinformatics.

[20] Osumi-Sutherland D, Costa M, Court R, O’Kane C. Virtual fly brain-using OWL to support the mapping and genetic dissection of the drosophila brain. In: C Maria Keet, editor. Proceedings of OWLED 2014. 2014. pp. 85–96. PMC5924869 29724079

[21] Virtual Fly Brain. In: Virtual Fly Brain [Internet]. [cited 30 Jun 2017]. Available:http://www.virtualflybrain.org

[22] Rector AL (2003). Modularisation of domain Ontologies implemented in description logics and related formalisms including OWL. Proceedings of the 2Nd international conference on knowledge capture.

[23] Li H, Horns F, Wu B, Xie Q, Li J, Li T, et al. Classifying drosophila olfactory projection neuron subtypes by single-cell RNA sequencing. bioRxiv. 2017:145045. 10.1101/145045. 10.1016/j.cell.2017.10.019 PMC6095479 29149607

[24] Euler T, Haverkamp S, Schubert T, Baden T (2014). Retinal bipolar cells: elementary building blocks of vision. Nat Rev Neurosci.

[25] Wilson RI (2013). Early olfactory processing in drosophila: mechanisms and principles. Annu Rev Neurosci.

[26] Gruber TR (1995). Toward principles for the design of ontologies used for knowledge sharing?. Int J Hum Comput Stud.

[27] Musen MA (2015). The ProtÉGÉ project: a look back and a look forward. AI matters. New York, NY. USA: ACM.

[28] Kazakov Y, Krötzsch M, Simančík F (2014). The incredible ELK. J Automat Reason.

[29] Chiang AS, Lin CY, Chuang CC, Chang HM, Hsieh CH, Yeh CW, Shih CT, et al. “Three-Dimensional Reconstruction of Brain-Wide Wiring Networks in Drosophila at Single-Cell Resolution”. Current Biology: CB 21. 2011;(1):1–11. 10.1016/j.cub.2010.11.056 21129968

